# Deciphering the Role of Multiple Thioredoxin Fold Proteins of *Leptospirillum* sp. in Oxidative Stress Tolerance

**DOI:** 10.3390/ijms21051880

**Published:** 2020-03-10

**Authors:** Daniela González, Pamela Álamos, Matías Rivero, Omar Orellana, Javiera Norambuena, Renato Chávez, Gloria Levicán

**Affiliations:** 1 Departamento de Biología, Facultad de Química y Biología, Universidad de Santiago de Chile, Avenida Libertador Bernardo O’Higgins 3363, Estación Central Santiago 917022, Chile; danigv.89@gmail.com (D.G.); pamela.alamos@gmail.com (P.Á.); matrivjim@gmail.com (M.R.); javis.cats@gmail.com (J.N.); renato.chavez@usach.cl (R.C.); 2 Programa de Biología Celular y Molecular, Instituto de Ciencias Biomédicas, Facultad de Medicina, Universidad de Chile, Santiago 8380453, Chile; oorellan@med.uchile.cl

**Keywords:** thioredoxin fold proteins, thioredoxin, oxidative stress, bioleaching, *Leptospirillum* sp. CF-1

## Abstract

Thioredoxin fold proteins (TFPs) form a family of diverse proteins involved in thiol/disulfide exchange in cells from all domains of life. *Leptospirillum* spp. are bioleaching bacteria naturally exposed to extreme conditions like acidic pH and high concentrations of metals that can contribute to the generation of reactive oxygen species (ROS) and consequently the induction of thiol oxidative damage. Bioinformatic studies have predicted 13 genes that encode for TFP proteins in *Leptospirillum* spp. We analyzed the participation of individual *tfp* genes from *Leptospirillum* sp. CF-1 in the response to oxidative conditions. Genomic context analysis predicted the involvement of these genes in the general thiol-reducing system, cofactor biosynthesis, carbon fixation, cytochrome *c* biogenesis, signal transduction, and pilus and fimbria assembly. All *tfp* genes identified were transcriptionally active, although they responded differentially to ferric sulfate and diamide stress. Some of these genes confer oxidative protection to a thioredoxin-deficient *Escherichia coli* strain by restoring the wild-type phenotype under oxidative stress conditions. These findings contribute to our understanding of the diversity and complexity of thiol/disulfide systems, and of adaptations that emerge in acidophilic microorganisms that allow them to thrive in highly oxidative environments. These findings also give new insights into the physiology of these microorganisms during industrial bioleaching operations.

## 1. Introduction

Thioredoxin (TRX) fold proteins (TFP) are a family of diverse proteins that possess a common domain consisting of four stranded beta-sheets flanked by three alpha-helices, and the redox-active CXXC motif [1]. The most representative member of this family is thioredoxin, a small disulfide reductase that helps to maintain a reducing cytosolic environment through reduction of oxidized thiols in cytoplasmic proteins [2]. The TRX fold has also been found in a number of other proteins, including the disulfide isomerases (DsbC, DsbD, and DsbG), disulfide oxidases (DsbA and DsbB), glutaredoxin, glutathione S-transferase, glutathione peroxidase, and peroxiredoxin [3]. 

TFPs share very little sequence homology and can be considered diverse in their functions and structures [4]. Like thioredoxins, many of these proteins are involved in thiol/disulfide exchange reactions, but also in oxidation and reduction reactions, depending on their redox properties. Thus, they play pivotal roles in protein folding and redox control in a number of biological processes. The wide range of redox activities of TFPs is possibly a consequence of modifications to the common scaffold, which result in different properties [5]. Thus, the role of these proteins cannot be directly deduced by the mere identification of a thioredoxin fold as structural feature. 

Oxidative stress, caused by reactive oxygen species (ROS), promotes disulfide bond formation in proteins. TFPs are thought to play an important function in response to this condition [4]. In addition to reducing cytoplasmic proteins, thioredoxins are indirectly involved in reducing endogenously- or exogenously-produced inorganic and organic peroxides. In this way, they provide reducing equivalents to thiol-based peroxiredoxins that are responsible for peroxide scavenging activity [6,7]. 

Thioredoxins also reduce oxidized redox sensors, thus regulating transcriptional activity of genes involved in physiological responses against oxidative stress [8,9]. In addition to thioredoxin and peroxiredoxin, TFPs include disulfide oxidoreductase [10], glutaredoxin [11,12], glutathione S-transferase [13], and glutathione peroxidase [14], all of which play important roles in the defense against oxidative stress. TFPs are thought to protect damaged proteins from inactivation and prevent aggregates of misfolded-protein through the reduction of disulfide-cross links, or through their formation in proper substrate proteins [15]. This latter fact is especially relevant for the folding, stability, and export of secreted proteins.

*Leptospirillum* spp. is an autotrophic obligate acidophilic bacterium that catalyzes dissimilatory Fe(II) oxidation. It serves as a model bacterium to elucidate the mechanisms used by acidophilic microorganisms to thrive under highly oxidative conditions [16,17,18,19]. Thirteen *tfp* genes (formerly *trx*) coding for predicted TFPs have been identified in *Leptospirillum* spp. [18]. Exposure of *Leptospirillum ferriphilum* to ROS-generating compounds resulted in a concomitant increase in thioredoxin activity. Interestingly, this activity was significantly higher than that measured in neutrophilic model bacteria. Since thioredoxin activity, measured through 1,4-dithiothreitol (DTT)-induced insulin, is present in most TFPs, the data suggest that proteins containing a TRX fold play a crucial role in the cell physiology and adaptation of *Leptospirillum* spp. to oxidative conditions. However, the specific contribution of each predicted TFP to the oxidative stress response and fitness in *Leptospirillum* spp. is still unknown. 

The goal of this work was to evaluate the individual participation of 13 *tfp* genes coding for TRX fold proteins from *Leptospirillum* sp. strain CF-1 in oxidative stress response. In order to deduce possible functions, we have used an approach that involved bioinformatic examination of the genomic neighborhood of the *tfp* genes. In addition, the expression patterns of the *tfp* genes were evaluated in strain CF-1 exposed to oxidative stress conditions. Finally, we established the ability of the genes to confer protection against oxidative stress to *Escherichia coli*, which is deficient in the thioredoxin system. 

## 2. Results

### 2.1. In Silico Analysis of the Genomic Context and Prediction of the Roles of the tfp Genes

A previous study demonstrated that the strain *Leptospirillum* sp. Group II ‘5-way CG’ possesses an extensive and diverse group of TRX fold proteins (TFPs) related to the cellular maintenance of the thiol/disulfide balance [18]. In this work, the genes for the 13 predicted TFPs from the 5-way CG strain were also detected in the genome of strain CF-1, and these predicted proteins were characterized bioinformatically. According to our results, the structural and functional features of these proteins are highly conserved among the two strains. For easier comparison, in this work we kept the numbering of the predicted proteins assigned in a previous paper (Table 1).

**Figure 1:**
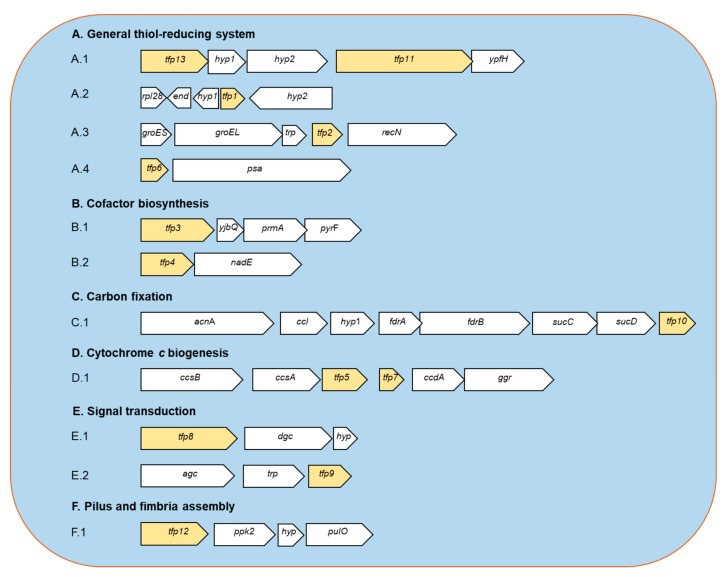
Predicted genetic context of TFP-encoding genes from *Leptospirillum* sp. CF-1. **A.1.** The *tfp13* gene codes for a: thioredoxin reductase (TrxB); *hyp1*: hypothetical protein 1; *hyp2*: hypothetical protein 2; *tfp11*: thioredoxin domain-containing protein (YyaL); *ypfH*: predicted esterase. **A.2**. *rpl28*: ribosomal protein L28; *end*: endonuclease; *hyp1*: hypothetical protein 1; *tfp1*: thioredoxin-like protein; *hyp2*: hypothetical protein 2. **A.3**. *groES*: co-chaperone GroES; *groEL*: chaperone GroEL; *trp*: TRP-containing small protein; *tfp2*: thioredoxin-like protein (chaperedoxin); *recN*: DNA repair protein RecN. **A.4**. *tfp6*: thioredoxin-like protein; *psa*: puromycin- sensitive aminopeptidase. **B.1.** *tfp3*: thiol disulfide oxidoreductase; *yjbQ*: thiamine synthase YjbQ; *prmA*: ribosomal protein L11 methytransferase PrmA; *pyrF*: Orotidine 5’-phosphate decarboxylase PyrF. **B.2.** *tfp4*: thioredoxin-like protein; *nadE*: NAD(+) synthetase. **C.1.** *acnA*: aconitase A; *ccl*: citryl-CoA lyase; *hyp1*: *fdrA*: fumarate reductase subunit A; *frB*: fumarate reductase subunit B; *sucC*: succinyl-CoA synthetase alpha subunit; *sucD*: succinyl-CoA synthetase beta subunit; *tfp10*: 2-Cys peroxiredoxin (Prx). **D.1.** *ccsB*: cytochrome *c*-type biogenesis protein CcsB; *ccsA*: cytochrome *c*-type biogenesis protein CcsA; *tfp5*: cytochrome *c*-type biogenesis protein CcsX; *tfp7*: thioredoxin-like protein; *ccdA*: cytochrome *c*-type biogenesis protein CcdA; *ggr*: geranylgeranyl reductase. **E.1.** *tfp8*: thiol disulfide oxidoreductase; *dgc*: GGDEF diguanilate cyclase; *hyp*: hypothetical small membrane protein. **E.2.** *agc*: adenylate/guanylate cyclase; *trp*: tetratricopeptide repeat (TRP)-containing protein; *tfp9*: thiol disulfide oxidoreductase. **F.1.** *tfp12*: cytoplasmic thiol disulfide oxidoreductase; *pkk2*: polyphosphate kinase 2; *hyp*: hypothetical protein; *pulO*: type IV prepilin peptidase.

Currently, a high-quality sequence of the entire genome of the CF-1 strain is available [20] (but not for 5-way CG). Given this, and in order to obtain information on the possible role of *tfp* genes and their encoded proteins, we analyzed the genomic context of each *tfp* gene (Figure 1). According to the collected data, TRX fold proteins can be assigned to six functional categories, namely the general thiol-reducing system, cofactor biosynthesis, carbon fixation, cytochrome *c* biogenesis, signal transduction, and pilus and fimbria assembly (Table 1, Figure 1). A brief description of the major features of each system is provided below.

#### 2.1.1. General Thiol-Reducing System (TFP1-TFP2-TFP6-TFP11-TFP13)

Thioredoxins are considered the primary system for maintenance of the intracellular thiol/disulfide balance. Moreover, in agreement with the previous report [16,18], no candidate genes related to glutathione system were detected in strain CF-1, which reinforces the idea that members of the *Leptospirillum* genus do not use glutathione as a redox buffer. Bioinformatic predictions suggest that strain CF-1 possesses a Trx system consisting of thioredoxin reductase TrxB (TFP13) and three Trx-like proteins (TFP1, TFP2, and TFP6); each of which has the canonical CXXC motif and is predicted to be located in the cytoplasm of the cell. According to an inspection of neighboring genes, the *trxB* is located next to genes for two hypothetical proteins, the uncharacterized conserved thioredoxin domain-containing protein YyaL (TFP11) and a putative esterase. The role of TFP11 remains unclear.

In order to gain insights into the possible roles of the three encoded thioredoxins, we compared them phylogenetically to several discrete types of thioredoxins from different sources. The results suggest that thioredoxins 2 and 6 from strain CF-1 are related to the prokaryotic/mitochondrial M-type thioredoxin class [21]. However, they comprise two discrete types, which suggests some degree of functional specialization (Figure S1). The TFP1 encoded protein is a thioredoxin-like protein with a low level of similarity to other characterized thioredoxins, and is grouped with thioredoxins from plants (types F, H, and O). No additional information could be derived from the genetic context of the *tfp1* gene. However, specialized functions related to protein folding and turnover could be predicted for TFP2 and TFP6, which have the canonical active site motif WCGPC found in TrxA and TrxC of *E. coli*, with a similar molecular weight (about 12 kDa) [22].

The GroESL protein complex in *E. coli* is the best characterized member of the HSP60/HSP10 heat shock chaperonin family and that is formed by chaperone GroEL and its obligate GroES co-chaperone [23]. GroESL is one of the major protein systems involved in folding newly-synthesized cytosolic proteins, and in refolding proteins that have been damaged under stress conditions. Thus, GroESL is believed to play a crucial role in the tolerance to all types of stress. Recent findings have shown that the GroESL system protects *E. coli* protein substrates during stress in cooperation with the chaperedoxin CnoX (chaperone-redox), which combines holdase activity with the ability to form mixed disulfide complexes with client proteins in an ATP-independent manner to prevent their aggregation [15].

Interestingly, CnoX is a thioredoxin-like protein that not only prevents protein aggregation, but also provides protection against irreversible oxidation by forming disulfide bonds with its substrates, and protecting their sensitive cysteines from over-oxidation. Due to this dual-function, CnoX has been assigned to a new family of proteins called chaperedoxins [15]. The substrates that are bonded to this chaperedoxin can only be transferred to foldase GroESL when intracellular reducing conditions are restored. Thus, this foldase assists the non-covalent folding of the protein in an ATP-dependent manner.

In *Leptospirillum* sp. CF-1, a putative thioredoxin-like protein (TFP2) gene is co-localized with *groEL* and *groES* genes (Figure 1A.3). Interestingly, TFP2 (but no other TFP) is highly similar (55%) to the N-terminal sequence (amino acids 1–66) of the CnoX protein from *E. coli* (284 aa). No other CnoX-encoding genes were detected in the genome from strain CF-1. Unlike CnoX from *E. coli*, TFP2 from *Leptospirillum* CF-1 possesses a CXXC motif and lacks the characteristic tretratricopeptide (TRP) repeat motifs [24]. However, a small open reading frame (ORF) in this genetic cluster that encodes for a predicted TRP-containing protein is located next to the *tf2* gene.

The cluster that harbors genes for GroEL, GroES, and the CnoX-like protein also contains a gene that encodes the protein RecN, which is predicted to be involved in the recombination repair of DNA double-strand breaks [25]. The organization of this genetic cluster is in agreement with a role of TFP2 in protein and DNA repair, so it may play a pivotal role in stress response in *Leptospirillum* sp. The role of TFP2 in *Leptospirillum* sp. as canonical thioredoxin and/or chaperedoxin should be explored experimentally.

Finally, the TFP6-encoding gene from the strain CF-1 is located close to a gene encoding for the putative puromycin-sensitive aminopeptidase PSA (or endopeptidase N from the M1 class peptidase) [26]. This enzyme is considered the major aminopeptidase in *E. coli* involved in cytosolic protein degradation, which enables the use of amino acids as nutrients [27]. The aminopeptidase encoded in CF-1 possesses 863 aa and has 35% similarity to the aminopeptidase N from *E. coli* (879 aa) [28]. The protein of the strain CF-1 has a predicted molecular weight of 97.9 kD, and conserves all the amino acid residues that have been reported as critical for the activity [29].

TFP6 showed a high degree of similarity to thioredoxin TrxC from *Rhodobacter capsulatus* (59%) and *E. coli* (54%), suggesting a role as a key antioxidant system through its disulfide reductase activity. Interestingly, TFP6 and aminopeptidase N-encoding genes are contiguous and very close, and could be part of a putative transcriptional unit. A functional relationship between TFP6 and aminopeptidase N linked to protein degradation can also be predicted. Redox-responsive protein degradation has been widely reported in different kinds of cells [30], so the redox status of the cell plays a key role in regulating protein turnover where oxidation has an inactivating proteolytic effect while reduction leads to an activation of proteolysis. Systems that reduce proteases include thioredoxins and glutaredoxins [30]. A relationship between putative thioredoxin TFP6 and aminopetidase N from *Leptospirillum* sp. CF-1 may be critical in determining redox-responsive enzyme activation and protein turnover.

#### 2.1.2. Cofactor Biosynthesis (TFP3 and TFP4)

The enzymes ThiE and thiamine phosphorylase ThiL catalyze the last two steps of the thiamine (vitamin B1) biosynthetic pathway. Interestingly, an analog (YbjQ) of the thiamine synthase ThiE has been found in many bacteria [31]. YbjQ has no structural similarity to ThiE, although members of the YbjQ protein family from several species have been shown to have thiamin phosphate synthase activity and complement *thiE* mutants [31].

In *Leptospirillum* sp. CF-1, visualization of the genomic neighborhood showed that the YbjQ-encoding gene clusters with three other genes that encode orotidine 5′-phosphate decarboxylase, methyltransferase, and thiol disulfide oxidoreductase (TFP3) (Figure 1B.1). According to our inspection, this cluster structure is only conserved in leptopirilli species. The biological significance of this genetic clustering should be studied. However, it may represent a relationship between disulfide oxidoreductase and thiamine biosynthesis. The involvement of the vitamin biosynthesis as part of oxidative response cannot be ruled out.

TFP4 is thought to be a membrane-anchored thioredoxin-like protein oriented towards the periplasm similar to the characterized thioredoxin-like protein TlpA protein (41% similarity) from *Bradyrhizobium japonicum* [32]. TlpA acts as a protein-disulfide reductase that has been shown to be physiologically significant [32]. The *tfp4* gene from *Leptospirillum* CF-1 was found next to the *nadE* gene (Figure 1B.2), which encodes for a glutamine-dependent NAD(+) cytoplasmic synthetase. NadE catalyzes ATP-dependent amidation of deamido-NAD in the last step of NAD(+) synthesis in both de novo and salvage biosynthetic pathways. The generated NAD(+) is an indispensable and universal redox cofactor involved in a variety of cellular processes. This dinucleotide is also a precursor of NADP+/NADPH, which is a key player in redox homeostasis maintenance in the cell [33].

The NADH:NAD(+) ratio is a significant indicator of the metabolic cellular state. It has been shown that intracellular concentration and the ratio of NAD(+)/NADH in *Bacillus subtilis* and *Mycobacterium tuberculosis* is modified in response to stress conditions [34]. Although cytoplasmic regulation of the NAD(+)/NADH ratio in chemolitoautotrophic bacteria is largely unknown, oxidative conditions may have an important effect in modulating the levels of this redox pair, so the synthesis of NAD(+) by the NadE enzyme may to some extent depend on the disulfide membrane reductase TFP4. Thioredoxin-mediated activation has been found in a number of enzymes [35], although redox activation has been not proven for NAD(+) synthetase. Thus, the relationship between this enzyme and disulfide reductase remains to be established.

#### 2.1.3. CO_2_ Fixation (TFP10)

Members of the *Leptospirillum* genus are obligate chemolitotrophic and autotrophic bacteria. CO_2_ fixation in these microorganisms seems to be performed by the reductive tricarboxylic acid (RTCA) cycle instead of the conventional Calvin–Benson cycle [36]. In *Leptospirillum ferriphilum* DSM14947, the enzymes for the RTCA cycle are encoded in four transcriptional units named *ccs* (for citryl-CoA synthetase), *ccl* (for citryl-CoA lyase), *for* (for 2-oxoglutarate ferredoxin oxidoreductase), and *por* (for pyruvate ferredoxin oxidoreductase) operons [36]. 

As in the strain DSM14647, the *ccl* operon of the strain CF-1 contains genes coding for aconitase A (*acn*A), citryl-CoA lyase (*ccl*), fumarate reductase (*fdr*AB), and succinyl-CoA synthetase (*suc*CD). Furthermore, this operon contains a gene that encodes for TFP10 that is thought to be a 2-Cys peroxiredoxin (Prx) belonging to the thioredoxin superfamily (Figure 1C.1). The Prx enzyme has been implicated in the reductive detoxification of peroxides [37]. Organic and inorganic peroxides are produced close to the cytoplasmic membrane during respiration and are strong oxidizing compounds that can damage carbon fixation proteins like aconitase [38], impairing the activity of the pathway. The activity of Prx can contribute to the maintenance of the cellular redox environment and consequently to the carbon fixation taking place.

#### 2.1.4. Cytochrome *c* Biogenesis (TFP5 and TFP7)

Biogenesis of *c*-type cytochromes involves the covalent attachment of the heme group to two cysteines present in apocytochrome *c,* and the subsequent folding of apocytochrome *c* around the heme. With this purpose, the prokaryotes use the pathways called Systems I, II, and III (extensively reviewed in Kranz et al., 2009) [39]. In general, attachment occurs on the external side of the cytoplasmic membrane and involves the formation of thioester bonds between the reduced cysteine residues of the CXXCH cytochrome motif and the alpha carbons of the heme vinyl groups [39]. The CXXCH cysteines are reduced, using a general transmembrane thioredoxin protein such as DsbD and specific thioredoxins such as CcmG of cytochrome *c* maturation system I or CcsX of cytochrome *c* synthesis system II. It has been reported that *Leptospirillum* sp. uses a plethora of *c-*type cytochromes that contribute to the maintenance of high respiratory rates during ferrous ion oxidation [40].

As noted in a previous report [18], our bioinformatic analysis in this work revealed that the genome of the strain CF-1 contains a cluster with genes that possibly encode for the CcsA, CcsB, CcsX, and CcdA proteins, which belong to System II of cytochrome *c* biogenesis. In addition to the gene that codes for the thioredoxin fold protein CcsX (TFP5), a second gene that codes for TFP7 was detected in this gene locus and annotated as a thioredoxin-like protein with a predicted membrane location. This protein is thought to contribute with reducing equivalents to restore the reduced state of CcsX or other target proteins.

Finally, it is interesting to note the presence of a geranylgeranyl reductase-encoding gene that may be involved in the structural modification of the hydrophobic side-chain of the heme molecule [41]. Hence, this locus seems to contain a complete set of genes related to the modification and trafficking of the heme group, and the biogenesis of type-*c* cytochromes. As described above, the participation in this process of two thioredoxin fold proteins (TFP5 and TFP7) was predicted.

#### 2.1.5. Signal Transduction (TFP8 and TFP9)

The second messenger cyclic diguanylate (c-di-GMP) has emerged in recent years as a central regulator for a number of bacterial processes, including biofilm formation, motility, and virulence [42]. c-di-GMP pathways have been reported in different *Acidithiobacillus* species. However, c-di-GMP effectors and signal transduction networks are still largely uncharacterized in this and other extremophile species. Interestingly, the involvement of c-di-GMP in biofilm formation on elemental sulfur in *Acidithiobacillus thiooxidans* was recently reported [43]. In *Leptospirillum* sp. CF-1, the existence of c-di-GMP pathways is suggested by the presence of genes that code for diguanilate cyclases GGDEF (with Gly-Gly-Asp-Glu-Phe at the active site) or GGEEF (with Gly-Gly-Glu-Glu-Phe at the active site), and EAL (Glu-Ala-Leu) or HD-GYP (His-Asp and Gly-Tyr-Pro) domain-containing phosphodiesterases, which are involved in synthesis and degradation of this molecule, respectively [44]. In this context, we were able to detect 12 genes coding non-identical versions of diguanylate cyclase in the genome of the strain CF-1, suggesting a role of these proteins in triggering cellular responses mediated by c-di-GMP under different environmental stimuli.

Similarly, to cyclic diguanylate, cyclic AMP (cAMP) is another important second messenger which is made by from adenosine triphosphate (ATP). In the vast majority of organisms, the formation and degradation of cAMP are mediated respectively by adenylyl cyclases and phophodiesterase, whose activity is controlled by glucose availability [45]. Changes in intracellular cAMP concentration are perceived by the cAMP receptor protein CRP. The cAMP-CRP regulatory coupling is able to respond to distinct external and internal inputs [45].

According to our bioinformatic inspection, TFP8 and TFP9 from the strain CF-1 are cytoplasmic thiol/disulfide oxidoreductases. The genetic context of the respective encoding genes revealed that they are located in two clusters that contain genes for a GGDEF domain-containing diguanylate cyclase (Figure 1E.1) and adenylate cyclase (Figure 1E.2) that may be directly involved in formation of c-di-GMP and cAMP, respectively.

The diguanylate cyclase (DGC) protein, encoded next to *tfp8* gene, possesses a transmembrane helix and a high isoelectric point, so this protein is possibly associated with the membrane and exposed to the acidic periplasm of the cell [46]. DGC has been shown to have a role in bacteria as a broad-range environmental sensor on the membrane [47]. The characteristics of the detected DGC from the strain CF-1 are consistent with a predicted role as a sensor. This genetic cluster also contains a gene that encodes for a small conserved protein of unknown function. However, according to bioinformatics, this protein has a predicted location in the cell membrane that also fits a possible role in the signaling pathway.

Disulfide oxidoreductase TFP8 codified in this genetic cluster is a soluble protein that may exert an antioxidant role in response to environmental assaults that generate oxidative stress. Interestingly, c-di-GMP has also been linked to the bacterial response to oxidative conditions [48]. Thus, a link between signaling pathways mediated by c-di-GMP and oxidative damage protection response could be established in the strain CF-1.

The TFP9-encoding gene is located close to genes for adenylate cyclase involved in cyclic AMP synthesis, and tetratricopeptide repeat-containing protein, as described in Figure 1. Tetratricopeptide repeats (TPRs) typically contain 34 amino acids and are found in proteins with a variety of functions [49]. To our knowledge, no relationship has been established between TRP proteins and the cAMP-dependent signaling pathway. However, it has recently been reported that the activity of regulating intracellular cAMP levels by the FimV protein is dependent on its C-terminal TRP domain in *Pseudomonas aeruginosa* [49]. Whether the activity of thiol-disulfide interchange activity of TFP9 is influenced by adenylate cyclase and TRP-containing protein or vice versa in *Leptospirillum* spp. is still an open question.

#### 2.1.6. Pilus and Fimbria Assembly (TFP12)

Phosphates can accumulate in cells as polymers of tens to hundreds of phosphate residues linked by inorganic phosphoanhydride bonds called polyphosphates (PolyPs). These polymers are synthesized by polyphosphate kinases (PPK) and degraded by polyphosphatases (PPX) [50]. PolyPs are produced by different *Acidithiobacillus* strains [51] and are believed to be present in other acidophiles.

There are two families of PPKs, type 1 (PPK1) and type 2 (PPK2). PPK1 from *E. coli* catalyzes the transfer of the terminal phosphate of ATP to a short polyP chain, but it can also catalyze the reverse reaction [50]. PPK2 is present in *Pseudomonas aeruginosa* and preferentially catalyzes the phosphorylation of GDP to support the biosynthesis of exopolysaccharides [52]. In the vicinity of *tfp12* there is a predicted *ppk* gene (Figure 1F.1) that encodes for PPK2 (51.74% homology to *Meiothermus ruber* PKK2). No PKK1 homologue was detected. The other interesting gene nearby in the genome encodes for a type IV prepilin peptidase (PulO) that is involved in the biosynthesis of pilus and secretion of substrates like toxins and enzymes that possess the IV pilin signal peptide [53].

According to our inspection, a complete type IV secretion system is not encoded in the CF-1 genome. Nevertheless, we detected genes in this strain related to the general secretory pathway (Gsp) that are clustered and form a putative operon *gsp*. Interestingly, the proteins encoded in this operon, which include the GspCDEF, and major pseudopilin GspG (NCBI accession number AKS23819.1 to AKS23827.1) are also necessary for the assembly of the type II secretion system [54]. Since most of the genes for the type IV secretion system do not appear to be present, the *pulO* gene that encodes a type IV prepilin peptidase and that is next to the TFP12 coding gene may have a role as the peptidase of the type II pilin. Our prediction suggests that the CF-1 genome contains a number of genes coding for possible target proteins of PulO, which typically contain a GFTLIE motif in the N-terminal region [55]. In agreement with this, bioinformatic predictions showed that target proteins include the pseudopilin GspG (AKS23823.1), among others.

In *E. coli*, periplasmic disulfide oxidase DsbA is required for the correct assembly of type IV fimbria that maintain the disulfide bond at the bundling C-terminal [56]. According to our inspection, TFP12 is a cytoplasmic thiol/disulfide protein, so activity similar to that of DsbA is unlikely. However, it may play a similar role in the CF-1 strain by maintaining proteins in proper redox and folding states for the further assembly of structures similar to those of pili or fimbria. Thus, we propose that protein products from *tfp12* and neighboring genes are involved in secretion and assembly of appendages, with TFP12 ensuring protein folding, PKK2 ensuring ATP supply required for transport of secreted proteins, and PulO cleaving signal sequences of necessary proteins (like GspG). TPL12 in *Leptospirillum* spp. may indirectly mediate many important functions where pilin/fimbria usually play pivotal roles (e.g., adherence to minerals).

### 2.2. The Expression of tfp Genes from Leptospirillum sp. CF-1 Is Differentially Regulated under Oxidative Stress Conditions

In order to determine the contribution of the predicted *tpf* genes to redox homeostasis, we determined whether oxidative stress elicitors increase the mRNA levels of each *tfp* gene. ROS overproduction and oxidative stress induction by exposure to ferric iron in *Leptospirillum* sp. had been assessed previously [18,19]. Diamide was used because of its ability to induce generalized disulfide stress. Cells were exposed for one hour to oxidative stress conditions with 260 mM ferric iron or 4 mM diamide. Changes in mRNA levels were estimated by real-time PCR and normalized against the 16S rRNA (*rrsB*) [19].

**Figure 2:**
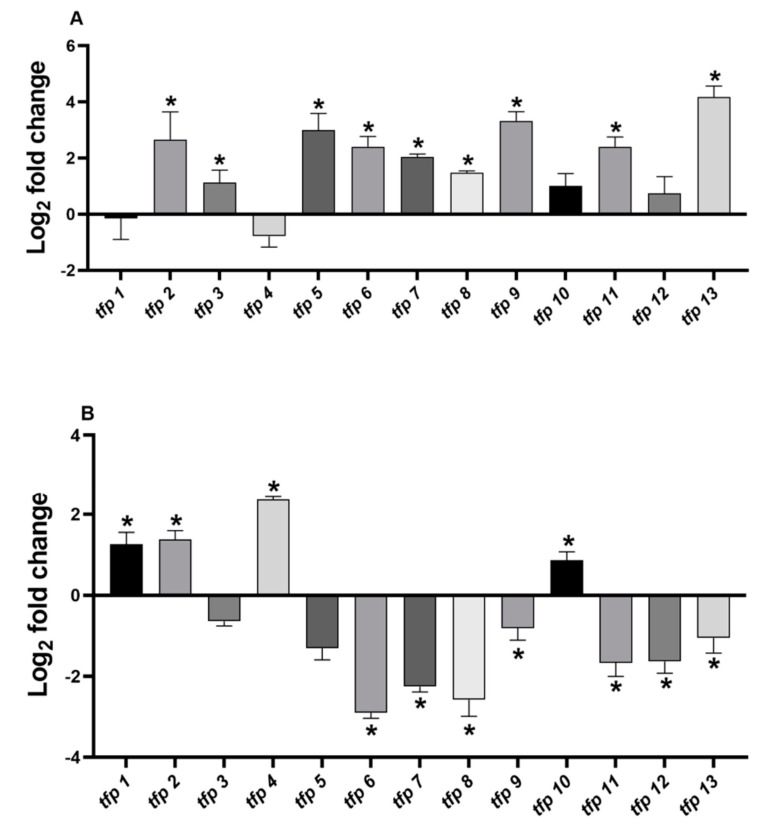
Relative expression of *tfp* genes from *Leptospirillum* sp. CF-1. Cells were stressed with (Fe_2_(SO_4_)_3_) (**A**) or diamide (**B**) for 1 h as indicated in Material and Methods. The RNA samples were analyzed in triplicate and ∆Ct results compared to the control 16S RNA gene. Bars represent the average of three independent experiments ± standard deviation. (*): *p* < 0.05.

Under ferric stress culture conditions, the genes *tfp2, tfp5, tfp9,* and *tfp13* were upregulated between 7- and 18-fold, while *tfp3*, *tfp6*, *tfp7*, *tfp8*, and *tfp11* showed increases of 2- to 5-fold (Figure 2A, Table S2) compared to control condition (without ferric iron). No significant changes were detected for *tfp1*, *tfp4*, *tfp10,* and *tfp12* mRNA levels.

The analysis of the relative mRNA levels of *tfp* genes in response to 4 mM diamide showed that *tfp1, tfp2, tfp4* and *tfp10* were upregulated between 2- and 5-fold, whereas *tfp6, tfp7, tfp8, tfp9, tfp11, tfp12* and *tfp*13 were downregulated, compared to the results under the control condition (without diamide) (Figure 2B, Table S2). No significant changes in mRNA levels were detected for *tfp3* and *tfp5*.

The results show that under ferric iron stress generalized upregulation of *tfp* genes took place (9 out of 13), whereas stress with diamide downregulated most *tfp* genes (7 out 13). The downregulation ranged from −1 to −2.9 log2 fold changes, representing decreases in mRNA levels of 50%–90%. For example, *tfp9* and *tfp*13 showed 10.2- and 18.5-fold change, respectively, in ferric iron stress, but in response to diamide they decreased by almost 50% (Table S2). Remarkably, one group of *tfp* genes was upregulated solely in response to ferric iron (*tfp3, tfp*5, *tfp*6, *tfp*7, *tfp*8, *tfp*9, *tfp11*, and *tfp13*), while another group was induced solely in response to diamide (*tfp*1, *tfp*4, and *tfp10*), suggesting that they are responsive to different stress signals. It is notable that *tfp2* increased in response to both ferric iron and diamide, which is in agreement with its possible role in preventing protein oxidation and aggregation.

It is also interesting to note that ferric iron, a naturally occurring stressor in bioleaching environments, produced a generalized upregulation and a likely activation of TRX fold proteins, including thioredoxin reductase TrxB (TFP13), as well as proteins thought to be involved in protein turnover (TFP2 and TFP6), thiamine biosynthesis (TFP3), biogenesis of cytochrome *c*, (TFP5 and TFP7), signaling, and disulfide bond isomerization (TFP8 and TFP9). Differences in the expression profile under ferric and diamide stress will be further discussed.

### 2.3. Tfp Genes from Leptospirillum sp. CF-1 Restored the Response to Oxidative Stress of a Thioredoxin-Deficient Strain of E. coli

Complementation assays were performed to determine whether *tfp* genes from *Leptospirillum* sp. CF-1 are functional and capable of conferring antioxidative protection to a Δ*trx*AΔ*trx*C thioredoxin-deficient strain (*E. coli* JEM-136). According to the expression profile determined by the RT-qPCR experiments, the following *tfp* genes were analyzed: *tfp2* and *tfp6* (upregulated in response to ferric ions)*, tfp1* and *tfp10* (upregulated in response to diamide), and *tfp12,* which was not responsive to ferric ions, but was downregulated under diamide stress. *E. coli trxA* (reported as constitutively expressed) encoding thioredoxin Trx1 from the wild-type strain K12 was used as a positive control.

**Figure 3:**
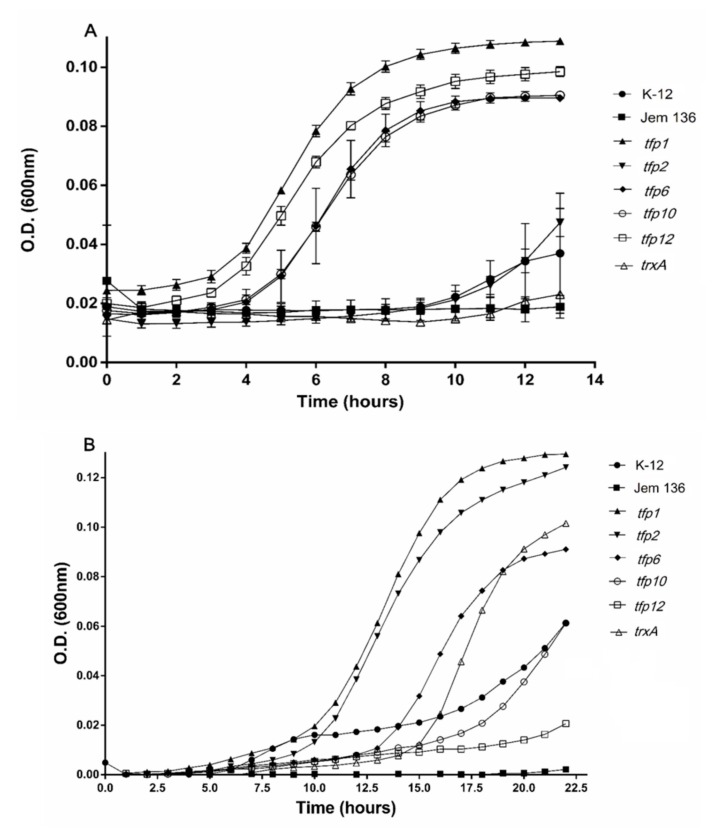
Growth of *Escherichia coli* JEM-136 complemented with *tfp* genes under oxidative stress culture conditions. Expression induction of the *tfp* gene in *E. coli* carrying pBADTopo-*tfp* was carried out with l-arabinose, as described in Materials and Methods. The complemented strain was grown on minimum tryptone yeast extract salts (TYES) medium under stress conditions with 0.5 mM (Fe_2_(SO_4_)_3_) (**A**) for 15 min, then transferred to a fresh medium, and grown for another 13 h. For diamide stress, the cells were exposed to 0.4 mM diamide and then grown for 20 h (**B**).

Initially, the susceptibility of *E. coli* ∆*trxAΔtrxC* mutant JEM-136 and the wild-type K-12 strains to ferric sulfate and diamide was assayed by determining their minimal inhibitory concentration (MIC). Sub-lethal levels of ferric iron (0.5 mM) and diamide (0.4 mM) (1/2 MIC) were used to evaluate the growth of the *E. coli* strains. As shown in Figure 3A, the strains transformed with the plasmid-carrying genes *tfp1, tfp6, tfp10,* and *tfp12* had higher growth rates with exposure to ferric ions than the wild-type strain, and the mutant JEM-136 strain transformed with an empty vector. These genes conferred a dramatically more notable protective effect than did *trxA* from *E. coli*. Interestingly, not only is the cell growth of strains carrying *tfp* genes higher than that of the control strains, but also the growth of complemented strains exposed to ferric iron were higher than those of control cells not exposed to the oxidant compound. For example, after 10 h of incubation under a ferric iron-stress condition, cells complemented with *tfp1*, *tfp12, tfp10,* or *tfp6* (induced with l-arabinose) had growth rates that were 67.5%, 63.7%, 57%, and 54.3% higher, respectively, than those of non-stressed cells (100%) (Table S3). In turn, growth of the mutant JEM-136 or the mutant strain complemented with *trxA* from *E. coli* K12 (TrxA-Ec) reached 15.3% and 25.7%, respectively. These data show that the *tfp1*, *tfp12, tfp10,* and *tfp6* genes from the *Leptospirillum* sp. strain CF-1 confer more protection to *E. coli* when exposed to oxidative conditions with ferric iron than does the endogenous gene *trxA*. As mentioned above, the TrxA protein from *E. coli* is a thioredoxin directly related to oxidative protection [7,57].

In the case of cultures exposed to diamide, the mutant strain JEM-136 complemented with predicted thioredoxin genes *tfp1* or *tfp2* had higher growth than the *trxA*-Ec-complemented strain or the wild-type K-12 strain, suggesting that these genes restore the phenotype to the thioredoxin-deficient strain, thus allowing this mutant to respond even more effectively to oxidative conditions than do wild-type strains (Figure 3B). Notably, after 10 h of incubation, higher percentages of growth recovery than under control condition (100%) were reached by strains complemented with *tfp1* (66.5%), *tfp2* (41.5%), and *tfp6* (10.5%), all of which are predicted to encode actual thioredoxins (Table S3). The strains complemented with the genes *tfp10* and *tfp12* did not have any noticeable growth stimulating effect under the oxidative condition.

Taken together, these results suggest that *tfp* genes from the strain CF-1 are functional and capable of restoring, and even improving the capability of *E. coli* to face stress conditions. In particular, *tfp1, tfp2*, and *tfp6*, which possibly encode thioredoxin-like proteins, restore and ameliorate the phenotype to a mutant *trx E. coli* strain in response to oxidative stress conditions. In addition to these genes, a significant protective effect was conferred by *tfp10*, which possibly encodes the 2-Cys peroxiredoxin Prx. In addition to their bioinformatic putative functions associated with regulation of the redox state, protein folding and turnover, and peroxide detoxification, these genes have a general protective role that is important under oxidative stress conditions. TFPs may contribute significantly to the fitness of *Leptospirillum* spp. in the extreme environments in which they inhabit and thrive.

## 3. Discussion

The reduction of disulfide protein bonds is mediated by a variety of thiol-redox enzymes that perform a rapid and reversible thiol/disulfide exchange. Members of the *Leptospirillum* genus lack the protein coding genes to generate glutathione and other thiol reductants such as glutaredoxins, or any known low-molecular-weight thiols. However, they have a predicted thioredoxin-based thiol/disulfide system composed of 13 coding genes.

The results presented in this research indicate that *tfp* genes found in *Leptospirillum* sp. CF-1 are transcriptionally responsive and their regulation is linked to oxidative stress, although they have differential expression depending on the oxidative agent used. Ferric iron resulted in the significant upregulation of nine *tfp* genes that are possibly linked to thioredoxins, cytochrome *c* biogenesis, cofactor biosynthesis, and signal transduction. *Leptospirillum* CF-1 seems to activate a number of genes encoding thioredoxin fold proteins from both cytoplasmic and membrane compartments to maintain the thiol/disulfide balance. The mRNA levels of the genes *tfp1*, *tfp2,* and *tfp6,* which possibly encode thioredoxins, significantly increased under ferric ion and/or diamide stress, and all were able to restore the growth of the mutant *E. coli*.

The deduced role of the gene products under oxidative stress is in accordance with the increased thioredoxin activity previously observed in cell extracts of the *Leptospirillum* strain 5-way CG in response to exposure to iron or diamide [18]. There was also a significant increase in the mRNA level of the gene *tfp13*, which is predicted to encode the NADPH-dependent thioredoxin reductase, TrxR. The role of this protein in providing reducing power to thioredoxins and peroxidases has been well-described, as has its essential function in the direct reduction of ROS and the acquisition of stress tolerance in a wide range of organisms [58,59]. It may also have an important role in oxidative protection in *Leptospirillum* spp. The *tfp10 and tfp12* genes, which possibly encode the 2-Cys Prx and a predicted cytoplasmic thiol/disulfide protein, were not upregulated when the strain CF-1 was exposed to ferric iron. Nevertheless, they allowed significant growth of the mutant *E. coli* strain when it was exposed to a sub-lethal concentration of ferric iron, which suggests these proteins have a protective role.

In contrast to the generalized upregulation of genes under ferric exposure, the mRNA levels of *tfp* genes in *Leptospirillum* CF-1 exposed to diamide stress indicate that only four genes were upregulated. Interestingly, the genes *tfp1* and *tfp2,* which were upregulated, were also functional in *E. coli* as they complemented the thioredoxin-deficient strain JEM-136. The results also suggest a discrete involvement of 2-Cys peroxiredoxin (TFP10) in disulfide stress defense. Strikingly, most of genes for thioredoxin fold proteins were not significantly affected by exposure to diamide stress. However, they may confer a significant level of protection to the thioredoxin-deficient *E. coli* strain. This protection was significantly higher than that conferred by the endogenous *trxA* gene.

In our previous study [18], the genes *tfp1*, *tfp6,* and *tfp13* of the strain 5-way CG were significantly upregulated by exposure to diamide, suggesting that this strain is more tolerant and active than the CF-1 strain when exposed to disulfide stress elicitors. A possible explanation to the reduced mRNA levels of these genes in response to diamide exposure is that this results from disulfide stress overcoming cell defense because of a generalized reaction of thiols to diazenecarbonyl diamide derivatives [60]. Consequently, cellular machinery for transcription, protein biosynthesis, and other metabolic pathways are negatively affected, thus impairing the ability of the cell to properly respond to extreme environmental conditions.

Exposure to ferric ions and diamide provides two models of oxidative stress induction [18], although they present noteworthy differences. While diamide triggers disulfide stress, ferric ions have a more direct oxidative effect that leads to their reduction and the consequent generation of ferrous ions, as well as the induction of Fenton chemistry, which in turn facilitates the generation of highly harmful radical species [17]. Additionally, oxidative stress was induced in this work with ferric iron in *E. coli* by exposure for 15 min and further growth was evaluated in fresh medium. Diamide was added and remained in the culture medium, which is why cells were constantly exposed to the oxidant.

Different stress induction protocols were applied because ferric ions tend to generate precipitates in neutral pH culture medium that reduce their bioavailability and alter the spectrophotometric measurement of growth. Although both agents induce oxidative stress, the mechanisms involved in this induction and the induced cell protection systems obviously differ to some extent. Despite this, the expression of these genes in one condition or another allows us to infer about their participation in the defense against ferric iron and diamide, as well as general redox protection.

An interesting question is why *tfp* genes from the strain CF-1 restore the wild type phenotype even better than the *trxA* from *E. coli*. In a previous work, we reported that crude cell extracts from *L. ferriphilum* showed higher thioredoxin activity than did *B. subtilis* and *E. coli* under either standard or diamide-induced stress conditions [18]. Furthermore, *E. coli* activity under the control condition was so low that it could not be detected in the extract under the test conditions. The results obtained here also support the view that thioredoxins from *Leptospirillum* spp. are more active and therefore more effective in dealing with oxidative stress. Highly redundant and complex thioredoxin systems in other microorganisms have been described [61,62]. Whether *Leptospirillum* spp. possesses high intracellular concentrations and/or high levels of specific Trx protein activity remains to be elucidated.

In addition, since there is no glutathione system, it is likely that thioredoxins from this microorganism play diverse roles in cellular metabolism, some of which may partially overlap with classic functions associated with glutaredoxins or glutathione in other organisms [63]. Finally, it should be noted that we were unable to detect canonical genes for the DsbA/B and DsbC/D systems, which have been widely described in a number of bacteria as participating in the oxidation and isomerization of thiols of extra-cytoplasmatic proteins, respectively [64]. *Leptospirillum* spp. may contain alternative systems for these functions. Therefore, a deeper characterization of the systems that govern the formation of disulfide bonds and increase stability of proteins at low pH is missing and needs to be assessed in this acidophilic bacterium.

In conclusion, the set of *tfp* genes that encodes for thioredoxin fold proteins in *Leptospirillum* sp. CF-1 is an active and functional system. Whether these proteins can successfully replace the role of the lacking glutathione or whether a different low-molecular-weight thiol contributes to redox homeostasis in this bacterium is still an open question. However, according to our results it should be assumed that the thioredoxin-based thiol/disulfide system plays an important role in oxidative stress response and the survival of *Leptospirillum* spp. under the highly oxidizing conditions imposed by acidic bioleaching environments.

## 4. Materials and Methods

### 4.1. Cell Cultures

*Leptospirillum* strain CF-1 cultures were grown aerobically at 37 °C and 180 rpm as described previously [19]. Bacterial growth was measured by direct microscopic counting using the improved Neubaüer chamber. *Escherichia coli* strains (DH5α, JEM-136, MG-1655) were grown in Luria Bertani (LB) or tryptone yeast extract salts (TYES) media at 37 °C and 150 rpm. Bacterial growth was estimated by measuring optical density (O.D.) at 600 nm, as indicated.

### 4.2. Induction of Stress in Leptospirillum sp. CF-1

One liter of *Leptospirillum* sp. CF-1 culture was grown to late exponential phase. Cells were harvested by centrifugation at 9000× *g* for 20 min at 15 °C and suspended in 25 mL of 9K-BR medium supplemented with 18.4 g/L ferrous sulfate (Fe_2_SO_4_). Cells were exposed to oxidative stress with 260 mM ferric iron (Fe_2_(SO_4_)_3_) or 4 mM diamide for 1 h at 37 °C and 150 rpm. Finally, cells were collected by centrifugation and washed with fresh medium.

### 4.3. RNA Extraction and cDNA Synthesis

Cells harvested from 1 L of *Leptospirillum* sp. CF-1 culture were suspended in 400 µL of lysozyme for 2 min (3 mg/mL in Tris EDTA (TE) buffer, pH 8.0. Later, 400 µL of lysis solution (20 mM sodium acetate pH 5.5, 1 mM EDTA, 0.5% SDS) and 400 µL of Trizol reagent (TRIsure Bioline, London, UK) were added. The samples were incubated for 10 min at 65 °C. The aqueous phase, obtained after centrifugation at 16,000× *g* for 5 min, was chloroform-extracted and ethanol-precipitated. Finally, the pellet was suspended in 30 µL of nuclease-free water. RNA integrity was electrophoretically analyzed. RNA concentration was estimated by spectrophotometry. The cDNA was synthesized using RevertAid reverse transcriptase (Thermo Fisher Scientific, Walthman, MA, USA) following the manufacturer’s instructions.

### 4.4. RT-qPCR and PCR

The reaction mixture contained 10 µL of 2× Fast SYBR Kapa, 0.4 µL of each primer (10 µM), 2 µL of cDNA, 0.4 µL 50× High ROX, and 6.8 µL of nuclease-free water. The primers are listed in Table S1. The qPCR conditions were an initial denaturation (95 °C for 20 s), followed by 40 cycles of denaturation (95 °C for 3 s), annealing (58 °C for 30 s) and extension (72 °C for 30 s). The reactions were performed in the StepOne Real-Time PCR system (Applied Biosystems, Foster, CA, USA). The relative abundance of the transcripts of the genes was determined using the constitutively expressed *rrsB* gene (16S rDNA) [19]. The experiments were carried out in triplicate.

### 4.5. Cloning of tfp Genes in an Expression Vector

The *tfp* genes were cloned in pBADTopo TA expression vector (Thermo Fisher Scientific, Walthman, MA, USA) to generate pBADTopo-*tfp*. The cloning was performed using the PCR product obtained from each *tfp* gene with genomic DNA from *Leptospirillum* sp. CF-1 as a template and specific primers (Table S1). The ligation product was obtained according to manufacturer’s instructions, and then introduced in *E. coli* TOP10 using the CaCl_2_ transformation protocol. To select transformants, the transformation mix was plated on media containing 100 µg/mL ampicillin.

### 4.6. Determination of Minimal Inhibitory Concentration

*E. coli* cultures grown overnight in minimum TYES medium were adjusted to McFarland standard 0.5, equivalent to 1.5 × 10^8^ cfu/mL. A range of concentrations was prepared for ferric sulfate and diamide solutions, and 100 µL were transferred to the first well of a 96-well microtitre plate and then serially diluted 1:1 with minimum TYES medium. Further, 50 µL of a bacterial culture were added. Cultures without oxidative stress elicitors, and TYES medium without cell culture were included as controls. The microplates were incubated overnight at 37˚C for 18 h and the absorbance at 600 nm was determined in an Elisa reader (Thermo Fisher Scientific Multiskan FC Model, Walthman, MA, USA).

### 4.7. Functional Assays of tfp Genes from the Strain CF-1 in E. coli

The recombinant plasmid pBADTopo-*tfp* gene was introduced into the *E. coli* strain JEM-136 (Δ*trx*AΔ*trx*C) by chemical transformation as indicated previously. To induce *tfp*-gene expression*,* 100 µL of an overnight culture of *E. coli* transformant were diluted to 10 mL of TYES minimum medium. Then, cells were grown at 37 °C to an O.D._600_ = 0.3, l-arabinose was added to a final concentration of 0.2% *w*/*v*, and the culture was incubated for another 5 h at 37 °C. Cultures treated with arabinose were diluted to obtain an O.D._600_ = 0.1, and then transferred to a 96-well plate and exposed to 0.4 mM diamide or 0.5 mM ferric iron as oxidative-stress elicitors [18]. For diamide stress, each well had 190 µL of TYES culture medium, 10 µL diamide 8 mM, and 2 µL of cell suspension. The growth was followed by measuring absorbance at 600 nm for indicated times. For ferric iron stress, cells were exposed for only 15 min to 0.5 mM Fe_2_(SO_4_)_3_ dissolved in a salt medium containing 0.5 g/L 7H_2_O·MgSO_4_, 0.5 g/L CaCl_2_·2H_2_O, 4 g/L NaCl, and 20 g/L glucose. The exposure to ferric ion was limited to avoid iron precipitation of ferric ions, which tends to occur spontaneously due to the neutral pH of the culture medium. Incubation was at 37 °C and 150 rpm, and ferric iron was removed by successive washing with a salt medium. Finally, cells were suspended in 200 µL of TYES medium and cellular growth was followed as indicated above.

### 4.8. Sequence Alignments and Motif Search

Amino acid sequences of enzymes predicted to possess thioredoxin fold motifs were obtained from the NCBI database. Direct comparisons were made using bioinformatic resources available in Expasy (https://www.expasy.org/). Multiple sequence alignments were performed using CLUSTALW and BLASTP. Putative motif and domains were determined using NCBI-CDD, Motif, and Motif Finder. The subcellular location was determined using ngLOC, CELLO v2.5, PSORTb, and Signal-P. Transmembrane domains were predicted with TMHMM v2.0.

### 4.9. Statistical Analysis

The data were analyzed through the unpaired student *t*-test using GraphPad Prism 8.0. A value of *p* < 0.05 was considered as a limit of significance.

## Figures and Tables

**Table 1:** Bioinformatic characterization of the predicted thioredoxin fold protein (TFP) of *Leptospirillum* sp. CF-1.

TFP	Accession Number	pI/MW	Cellular Location	Protein	Genomic Context-Derived Function
1	WP_023525652.1	9.47/9512.98	ND	Thioredoxin-like	General thiol-reductase system
2	WP_014960847.1	5.42/11712.63	Cytoplasm	Thioredoxin-like	General thiol-reductase system
3	WP_023524535.1	7.73/38929.40	ND	Thiol disulfide oxidoreductase	Thiamine biosynthesis
4	WP_023525480.1	9.30/23548.14	Cytoplasmic membrane	Thioredoxin (Trx2)-like proteins	NAD+ biosynthesis
5	WP_014962035.1	9.08/22475.37	Cytoplasmic membrane	Thioredoxin fold protein CcsX	Cytochrome *c* biogenesis
6	WP_036080148.1	5.70/17114.98	Cytoplasm	Thioredoxin-like	General thiol-reductase system
7	WP_049713760.1	8.60/12904.08	Cytoplasmic membrane	Thioredoxin-like protein	Cytochrome *c* biogenesis
8	WP_023524407.1	5.34/4016.03	Cytoplasm	Thiol/Disulfide oxidoreductase	Signal transduction
9	WP_042225252.1	5.68/24829.29	Cytoplasm	Thiol/Disulfide oxidase	Signal transduction
10	WP_014960570.1	6.82/17281.80	Cytoplasm	2-Cys peroxiredoxin	CO_2_ fixation
11	WP_053094110.1	5.56/79330.91	Cytoplasm	Thioredoxin domain-containing protein YyaL	General thiol-reductase system
12	WP_038505022.1	6.18/25842.79	Cytoplasm	Cytoplasmic thiol disulfide oxidoreductase	Pilus/fimbria assembly
13	WP_014962072.1	5.91/33482.30	Cytoplasm	Thioredoxin reductase TrxB	General thiol-reductase system

ND: Not Determined.

## Supplementary Materials

Supplementary materials can be found at https://www.mdpi.com/1422-0067/21/5/1880/s1.
