# Supplementary material for: Deciphering the Role of Multiple Thioredoxin Fold Proteins of Leptospirillum sp. in Oxidative Stress Tolerance
Source: Int J Mol Sci. 2020 Mar 10;21(5):1880. doi: 10.3390/ijms21051880 (PMC7084401; doi:10.3390/ijms21051880)
Supplement: Supplementary file 1 [file ijms-21-01880-s001.zip › ijms-748915 suppl for final/Table S1,S2,S3.pdf]

Table S1. Oligonucleotides used in this work.

Target	Name	Sequence 5´-3´	Product Length (bp)
*tfp1**	pTfp1-F	AAGGTTTCGGTCCTCTATGC	151
	pTfp1-R	TGAATGGAGTGACGTTGAGC	
*tfp2**	pTfp2-F	CTGGTAATGGTTGATTTCTGGG	104
	pTfp2-R	GGAATGCCCATGACCTGATAT	
*tfp3**	pTfp3-F	TGTTCTGATCCGGAAAAACC	184
	pTfp3-R	GTCGACCAGAACGACTTTCC	
*tfp4**	pTfp4-F	CTGGATTTGGGAAAACTGC	113
	pTfp4-R	TCTATTTGACGGGACAGACG	
*tfp5**	pTfp5-F	CATTGATCAGAATGGTGTGG	109
	pTfp5-R	CGTCTTTGGTCCTTTGTC	
*tfp6**	pTfp6-F	GACGGACGACACCTTTCAGA	138
	pTfp6-R	AGTCTCCCGTGGTACTTTCG	
*tfp7**	pTfp7-F	AGTTTGAGAGCCGTGAAGC	163
	pTfp7-R	AAGCCGTGAACTTTGTATGC	
*tfp8**	pTfp8-F	AAGAAAAATATCCCGACAGC	107
	pTfp8-R	GATGGCATAACGTTCAATGG	
*tfp9**	pTfp9-F	CGATTTTCTCTCCGCGATGA	81
	pTfp9-R	GCAATGTCTTTCAGAACGGG	
*tfp10**	pTfp10-F	TATCCGAGAGAAGGTTGTGC	119
	pTfp10-R	TGATCTTCCCAAGTTTGACC	
*tfp11**	pTfp11-F	AGAGTCTGCAGGACAGAAGC	138
	pTfp11-R	TCCTGCTTTCCAAGAATACG	
*tfp12**	qTfp12-F	TCGAAACGGCAGATAATACCT	80
	qTfp12-R	TTTGACCTGACCACCGACAA	
*tfp13**	ptfp13-F	TTGCGATTCGGGACCC	108
	ptfp13-R	CTCCRCCAACGACMACG	
pBadTOPO	pBadTopo-R	GATTTAATCTGTATCAGGCTG	335
	pBadTopo-F	ATGCCATAGCATTTTTATCCAT	
*rrsB**	pRrsB-F	TACAAGCTTCCGCTCCTG	288
	pRrsB-R	CCGGGCAAAAGTGGTTTACA	
*tfp1**	pBad_Tfp1-F	ATGGCCAAGGTTTCGGTC	252
	pBad_Tfp1-R	CTTAAGCGTTTTCAGGAGGTG	
*tfp2**	pBad_Tfp2-F	GTGGAAGTAAATGCTCCGGA	318
	pBad_Tfp2-R	GGACTTGAGAAGAGAGTCAAT	
*tfp6**	pBad_Tfp6-F	ATGGATGAGGATATTCTGATGA	471
	pBad_Tfp6-R	GGAAGGGAGCGGGCTGCT	
*tfp10**	pBad_Tfp10-F	ATGGCTGCTGAAATAAAAGTGGG	462
	pBad_Tfp10-R	CTATTTCAACTGTTCAAGCGC	
*tfp12**	pBad_Tfp12-F	ATGCAAAAGGTCCCATTT	765
	pBad_Tfp12-R	CTACTTCTTTTGTCCGAG	
*tfpA***	pTfpA-F	ATGAGCGATAAAATTATTCACCT	330
	pTfpA-R	TTACGCCAGGTTAGCGTC	

(*) *Leptospirillum* sp. CF-1 ; (**) *E. coli* K-12.

Table S2. Relative fold change in mRNA levels of *tfp* genes from *Leptospirillum* sp. CF-1 exposed to oxidative stress with ferric ion or diamide.

Gene	Ferric Ion	Diamide	
	Increase	Increase	Decrease
tfp1	-	2.43 ± 0.48	
tfp2	7.33 ± 4.74	2.62 ± 0.39	
tfp3	2.25 ± 0.69	-	-
tfp4	-	5.17 ± 0.24	

tfp5	8.44 ± 3.38	-	-
tfp6	5.4 ± 1.36		0.13 ± 0.01
tfp7	4.12 ± 0.29		0.21 ± 0.02
tfp8	2.78 ± 0.14		0.17 ± 0.05
tfp9	10.2 ± 2.27		0.57 ± 0.12
tfp10	-	1.84 ± 0.26	
tfp11	5.38 ± 1.30		0.32 ± 0.07
tfp12	-		0.33 ± 0.07
tfp13	18.51 ± 4.95		0.49 ± 0.13

(-), no significant changes.

Table S3. Percentage of growth recovering of stressed respect to non-stressed cultures of *tfp*-complemented cells.

Strain	Ferric Ion	Diamide
E. coli K-12	14.20 ± 2.10	12.62 ± 0.77
Jem-136	15.29 ± 3.25	1.35 ± 0.15
Jem-136 / *tfp1*	67.50 ± 1.10	66.48 ± 12.26
Jem-136 / *tfp2*	21.47 ± 1.36	41.48 ± 1.76
Jem-136 / *tfp6*	54.28 ± 0.32	10.47 ± 1.01
Jem-136 / *tfp10*	56.70 ± 0.58	9.30 ± 1.15
Jem-136 / *tfp12*	63.70 ± 0.75	6.92 ± 0.54
Jem-136 / *trxA*	25.69 ± 2.02	5.69 ± 1.04

Growth of non-stressed cells was considered 100%. JEM-136 genotype: *ΔtrxAΔtrxC*.
