# Supplementary figures and images for: Deciphering the Role of Multiple Thioredoxin Fold Proteins of Leptospirillum sp. in Oxidative Stress Tolerance
Source: Int J Mol Sci. 2020 Mar 10;21(5):1880. doi: 10.3390/ijms21051880 (PMC7084401; doi:10.3390/ijms21051880)

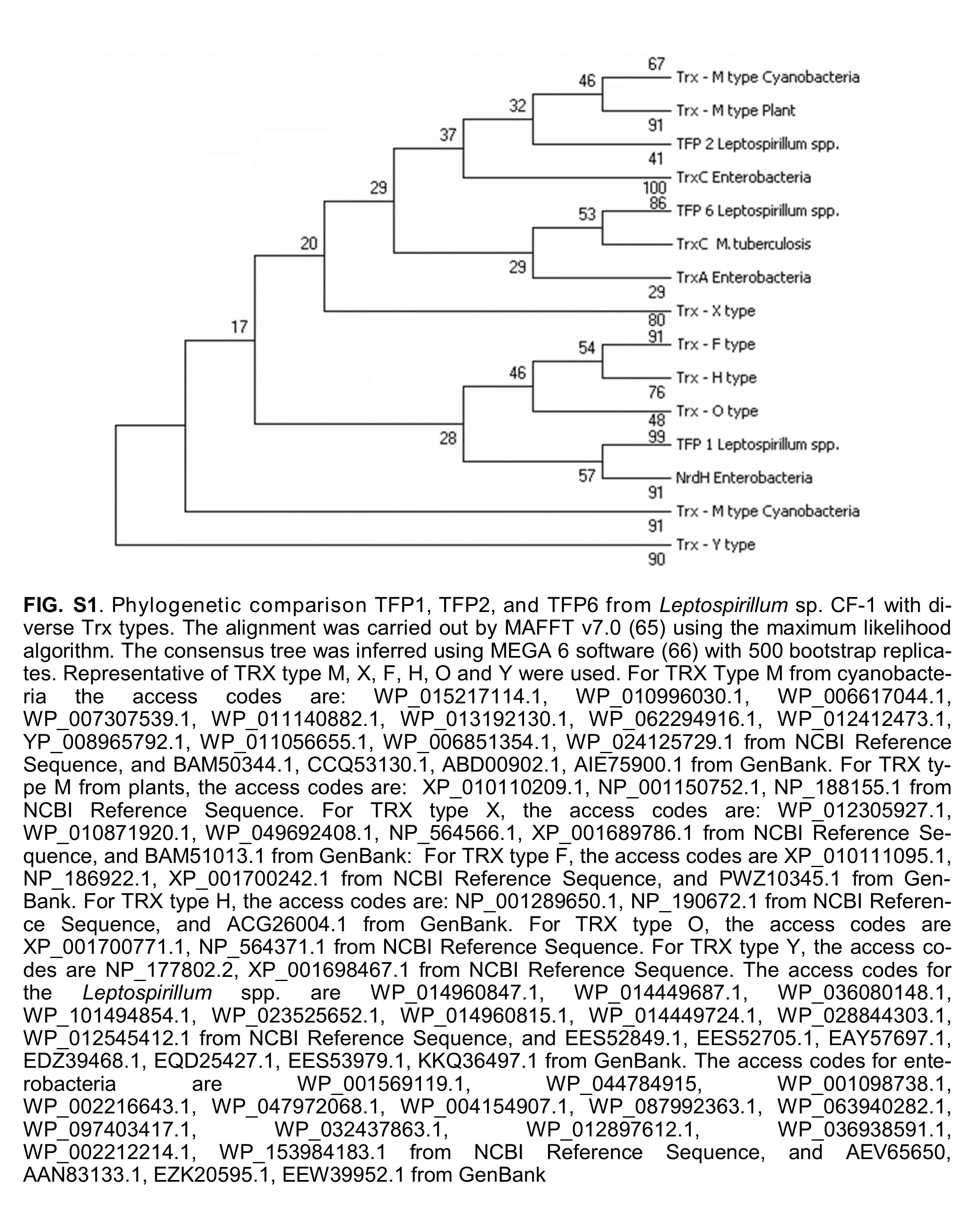

Supplement: Supplementary file 1 [file ijms-21-01880-s001.zip › ijms-748915 suppl for final/Figure S1.tif]
